# A New Blondin System for Surveying and Photogrammetry

**DOI:** 10.3390/s131216894

**Published:** 2013-12-06

**Authors:** Federico Cuesta, Francisco M. Lopez-Rodriguez, Antonio Esteban

**Affiliations:** 1 Escuela Técnica Superior de Ingeniería, University of Seville, Camino de los Descubrimientos s/n, Seville E-41092, Spain; E-Mail: franciscom.lopez.rodriguez.edu@juntadeandalucia.es; 2 Sacyr S.A.U., Av. Luis Montoto 107, Seville E-41007, Spain; E-Mail: aesteban@sacyr.com

**Keywords:** sensors integration, surveying, construction site, cascaded extended Kalman filter (EKF), structure from motion (SfM), dense point cloud, digital surface model (DSM)

## Abstract

The main objective of the system presented in this paper is to provide surveyors and engineers with a new photogrammetry device that can be easily integrated with surveying total stations and a global navigation satellite system (GNSS) infrastructure at a construction site, taking advantage of their accuracy and overcoming limitations of aerial vehicles with respect to weight, autonomy and skilled operator requirements in aerial photogrammetry. The system moves between two mounting points, in a blondin ropeway configuration, at the construction site, taking pictures and recording the data of the position and the orientation along the cable path. A cascaded extended Kalman filter is used to integrate measurements from the on-board inertial measurement unit (IMU), a GPS and a GNSS. Experimental results taken in a construction site show the system performance, including the validation of the position estimation, with a robotic surveying total station, or the creation of a digital surface model (DSM), using the emergent structure from motion (SfM) techniques and open software. The georeferencing of the DSM is performed based on estimated camera position or using ground control points (GCPs).

## 1. Introduction

Advances in sensors technologies have had a significant impact during the last decades on the ground-level tools used by surveyors and construction engineers in their daily tasks, including surveying, layout and grade management of a construction project [1]. Thus, nowadays, devices with millimetric or centimetric accuracy, such as digital theodolites, total stations, robotic total stations, terrestrial laser scanners (TLS) or real-time kinematics global navigation satellite systems (RTK-GNSS), are common tools on any construction site. Recently, Gikas [2] showed the benefits and limitations of using robotic total stations and TLS technology, to collect high-fidelity data to support tunnel construction in two construction sites in Greece. Park *et al.* [3] developed a monitoring system for complex building construction using a laser displacement sensor (LDS) and wireless sensors, whose accuracy and reliability were verified with a total station. Siu *et al.* [4] used two robotic total stations and three cameras to provide a solution to monitor the dimensional changes of a temporary facility in the construction field. In [5], El-Omari and Moselhi make use of a 3D laser scanner and photogrammetry, in an integrated way, to collect data from construction sites and to compare actual work to planned work in a building under construction. Fregonese *et al.* [6] analyzed the use of TLS and total stations for the surveying and monitoring of civil construction and historical buildings.

Improvements become evident not only for ground-level devices, but also for airborne-based tools, especially in aerial photogrammetry, mainly due to advances in digital images, CMOScamera sensors, MEMS inertial measurement units (IMU), integrated IMU-GPS devices, laser sensors, *etc.* Many applications have been reported about using airborne devices [7], including aerial mapping with handheld sensors [8], land surveying of construction sites [9], automated determination of 3D city models from airborne acquired point cloud data [10], cadastral applications [11], forest inventory [12], photogrammetric surveys in archaeological areas [13] or the generation of georeferenced digital terrain models based on structure from motion approaches [14].

Several types of flying objects, such as conventional planes, helicopters [8], kites [15] or a wide variety of unmanned aircraft systems (UAS) [7,16,17], had been used. At this point, UAS deserve special attention (see [16,17] and the references therein for a complete classification and considerations of use). Among them, micro UAVs, which require no takeoff or landing run, have experienced a significant spread in the last decade, mainly boosted by lightweight, low-cost MEMS-based IMU, GPS, cameras or lasers. The counter-part of these low-cost devices is a reduction of accuracy and performance, which can be mitigated by using sensor fusion techniques for pose estimation (both position and orientation) [12,18,19]. On the other hand, these vehicles usually have a reduced payload (lower than 3 kg) and limited autonomy. UAVs with accurate on-board devices, augmented payload and autonomy can also be found, but at a significantly higher cost [16]. Another key point is that these vehicles usually require a skilled operator, either to remote control or as the safety pilot in the case of UAVs with autopilot systems (in some cases, the autopilot can only be activated when the vehicle is in the air, but take-off and landing have to be operated manually) [16].

**Figure 1.**
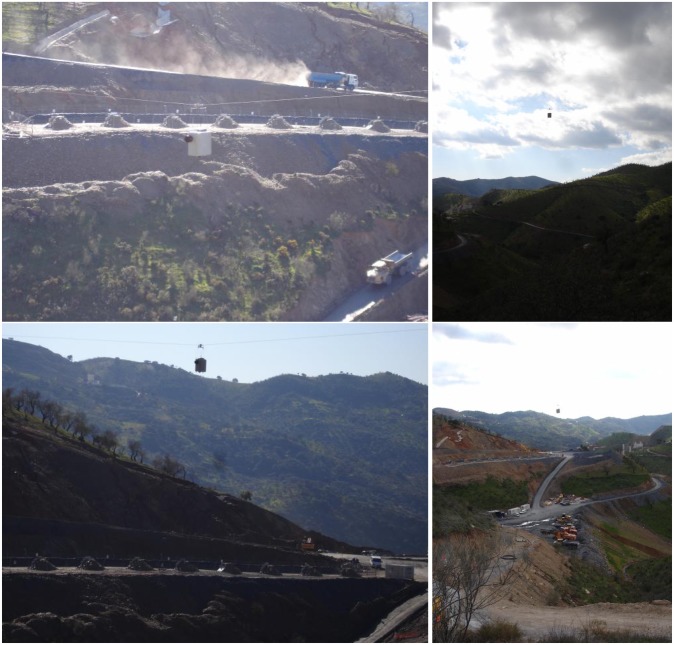
System at the construction site.

This paper presents a new system designed to take advantage of ground device accuracy (facilitating the integration with surveying total stations, RTK-GNSS and the construction site infrastructure) and, at the same time, overcoming the limitations of aerial vehicles with respect to weight, autonomy and the need for skilled operators. Thus, the system integrates an accurate RTK-GNSS, commonly used at construction sites, a prism for interfacing with a robotic total station, a high resolution camera and a medium-cost IMU into a device, weighing around 12 kg in total. It moves between two mounting points, in a blondin ropeway configuration, at the construction site, taking pictures and recording the position and orientation data along the cable, as shown in Figure 1. Using two mounting points can be a disadvantage with respect to the coverage area, but this could be solved by using a Skycam configuration. The prototype has been developed in a research project funded by the Spanish construction company, Sacyr SAU.

This paper is organized as follows: Section 2 describes the sensors on board the system, as well as their integration using a cascaded extended Kalman filter. Section 3 shows some experimental results at a construction site, including the validation of position estimation, with a robotic surveying total station, or the creation of a digital surface model (DSM), using structure from motion (SfM) techniques and open software. Georeferencing of the DSM is performed based on the estimated camera position or by means of ground control points (GCPs). Section 4 ends with the conclusions.

## 2. System Description and Sensors Integration

**Figure 2.**
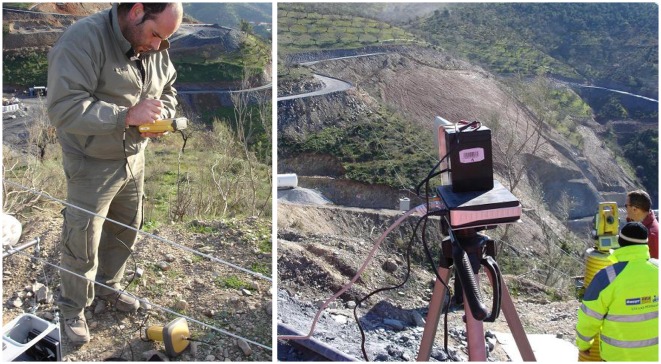
Surveyor at work configuring a conventional Topcon GR-3 real-time kinematics global navigation satellite system (RTK-GNSS) unplugged from the system **(left)** and recording the system position by using a robotic total station **(right).**

As mentioned above, the main objective of the system presented in this paper is to provide surveyors and engineers with a new photogrammetry device that can be easily integrated with the surveying total stations and GNSS infrastructure at the construction site. Thus, by including in the system an omnidirectional prism and a commonly used centimetric RTK-GNSS, it is possible to take advantage of the high precision provided by the total stations and available GNSS corrections. Moreover, conventional equipment facilitates the usage and integration of surveying operators (see Figure 2).

From a functional point of view, the system is fixed to a cable in such a way that it can move between two mounting points, in a blondin ropeway configuration, taking pictures and recording data of the position and orientation along the cable path. The system is connected to a remote PC, via a directional WiFi antenna (see Figure 2 (right)), for telemetry data logging and camera recording.

**Figure 3.**
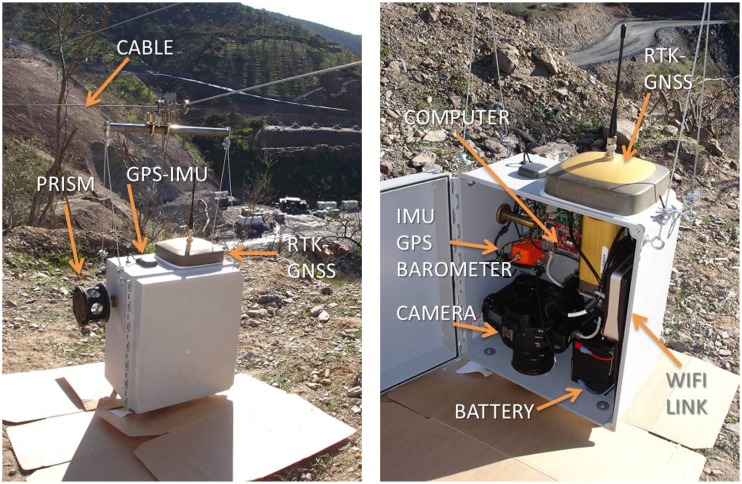
Sensors on board the system.

The system has a wide variety of sensors (see Figure 3), including 3D-accelerometers, 3D-rate gyroscopes, 3D-magnetometers, barometer, thermometer and GPS, all of them integrated into a commercial medium-low cost IMU-GPS unit (Xsens MTI-G), as well as a high resolution RTK-GNSS (Topcon GR-3), a high resolution professional photo camera (Canon EOS-1Ds Mark III), a 360 degree prism and a Wi-Fi link. An embedded computer collects and integrates data from all the sensor devices, and it also connects with the remote PC. Sensors are enclosed in an IP66compliant box, for protection against dust and water. The total weight of the system (batteries included) is around 12 kg, with an autonomy of several hours.

Concerning the IMU-GPS, it is a MEMS-based unit (with solid state capacitive readout accelerometers and rate of turn sensors and a silicon bulk micro-machined pressure sensor), which is able to provide heading and attitude with a dynamic accuracy of three and 1° RMS, respectively (the static accuracy is lower than one and 0.5°). The angular resolution is 0.05°. On the other hand, the GPS position accuracy is 2.0 m CEP (Differential GPS mode). The 3D magnetometer and barometer are used in an internal real-time Kalman filter as aiding sensors to directly observe magnetic north (improving heading estimation) and to account for varying pressure (which affects altitude estimation).

On the other hand, Topcon GR-3 is an advanced GPS+ (triple constellation GPS, GLONASS, GALILEOreceiver), specially designed for surveying and engineering applications. In real-time RTK mode, the accuracy is 10 mm + 1 ppm (horizontal) and 15 mm + 1 ppm (vertical). It should be pointed out that the static accuracy can be improved with post-processing up to +3.0 mm + 0.5 ppm (horizontal) and +5.0 mm + 0.5 ppm (vertical). In differential mode, the accuracy is lower than 0.5 m in real time (and higher than 0.25 m with post-processing). Obviously, it is a high cost unit.

The on-board camera (Canon EOS-1Ds Mark III with a Canon EF 14 mm f/2.8L II USMUltra-Wide Angle Lens) is a medium−high cost, professional digital camera, with a 21.1 Megapixel image sensor. Images can be obtained in raw format with a resolution of 5, 616 × 3, 744 pixels. An effective sensor size is 36 × 24 mm, and the lens focal length, 14 mm.

## 2.1. Sensors Integration and Extended Kalman Filter

Sensor integration is paramount for the system performance, *i.e.*, to accurately estimate both position and orientation. This makes it mandatory to use a sensor fusion algorithm (such as a Kalman filter or a similar one [12,18-20]) and to be also very careful with data formats and with the definition of the reference frames (especially with GPS measurements from different devices) [21].

**Figure 4.**
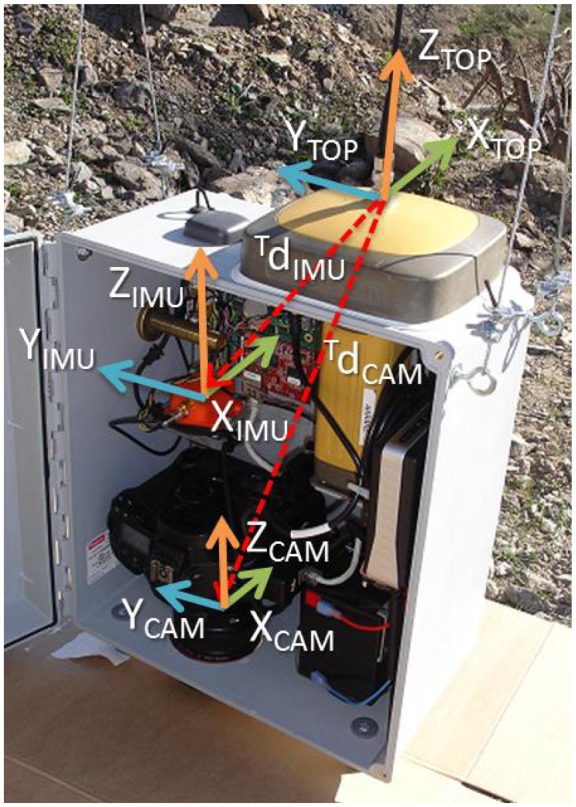
Local reference frames.

To start with, we have to define the different local reference frames, as well as their relations in terms of translations and rotations [20]. In this way, the origins of four frames will be defined, *O_TOP_*, *O_IMU_*, *O_CAM_* and *O_PRI_*, corresponding to the Topcom GNSS, IMU, camera and prism, respectively. However, to define the frame, orientation is also needed. By default, the IMU local reference frame is aligned to its external housing, with *X_IMU_* pointing forward, *Y_IMU_* pointing left and *Z_IMU_* pointing upward, as shown in Figure 4. Taking into account that orientation with respect to the global world frame is provided by the IMU, sensors were placed within the box in such a way that rotations were avoided. Therefore, the orientation of frames *TOP, PRI* and *CAM* was defined as having the same orientation as the *IMU* frame. Finally, translation vectors from a reference frame to another have to be defined and accurately measured. For instance, Figure 4 depicts vector $d_{\text{IMU}}^{\text{TOP}}$, which stands for the position of *IMU* in the reference frame, *TOP*.

**Figure 5.**
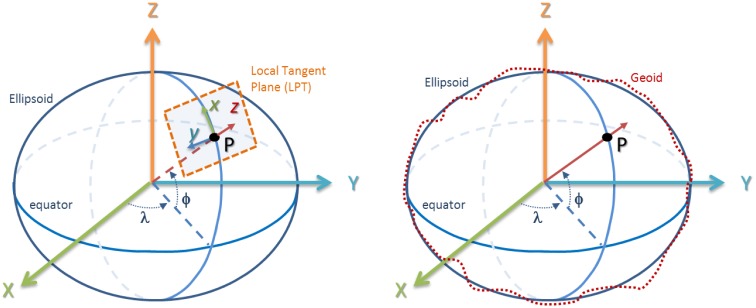
The Earth-Centered Earth-Fixed (ECEF) coordinate system and Local Tangent Plane **(left)** and ellipsoid *vs.* Earth geoid **(right).**

The next step is to select a global reference frame. Since we are dealing with positioning, it is preferred to use an Earth-Centered Earth-Fixed (ECEF) coordinate system that rotates with the Earth [21]. In the ECEF system, the *XY* plane coincides with the equator, the *Z-*axis points to the north and the X-axis points to the Greenwich meridian (0° longitude), as shown in Figure 5. However, it is difficult to relate global coordinates of a point in an *XYZ* ECEF reference system with a map of Earth's surface. To solve this problem, it is possible to use a physical model of the Earth, such as the World Geodetic System 1984 (WGS84), which is the standard for GPS applications, to define a point in terms of latitude, longitude and altitude, *i.e.*, in a spherical coordinate system [21].

In WGS84, Earth's shape is approximated by an ellipsoidal model defined by several parameters (semi-major and semi-minor axes, inverse flattering, *etc.).* Because this could be rather generic, accuracy can be improved by using local models (called datums), which are, basically, different values for the ellipsoid axes and center [20]. The GPS receiver integrated with the IMU uses the generic WGS84 datum.

Even more, the spherical coordinate system of WGS84 can be transformed into a Cartesian coordinate system by means of a linearization of the WGS84 ellipsoid, yielding a local tangent plane (LTP) (see Figure 5), with *X_LTP_* pointing north, *Y_LTP_* pointing west and *Z_LTP_* pointing upward.

Another, commonly used Cartesian coordinate system is the Universal Transverse Mercator (UTM), which is also based on the WGS84 ellipsoid to model the Earth [21]. UTM divides the surface of the Earth into sixty zones (called UTM zones) and gives locations in a 2D Cartesian coordinate system, with the *X*-axis pointing east and the *Y-*axis pointing north. It is also known as the easting-northing coordinate system. Notice that UTM implies a 90-degree rotation around Z with respect to LTP, which is a northing-westing description.

Finally, it should be remarked that GPS altitude can be measured above the ellipsoid or above mean sea level (MSL) [20]. Both altitudes can be quite different, since there are significant differences between the ellipsoid and Earth geoid (see Figure 5 (right)). By default, Xsens GPS provides altitude above the ellipsoid. However, Topcon GR-3 GNSS uses the National Marine Electronics Association (NMEA) standard, which deals with altitude above the geoid. Fortunately, it also provides the geoidal separation, *i.e.*, the difference between the Earth ellipsoid and geoid defined by the reference datum. Altitude above the ellipsoid can be then obtained by adding MSL altitude and geoidal separation (which can be negative). In this system, NMEA altitude will be used as reference, so geoidal separation will be subtracted from the Xsens GPS altitude. In the experiments shown in Section 3, the geoidal separation was 48.776 m.

On the other hand, we use the IMU to measure a global orientation. The problem here is that inertial measurements should be referred to as an Earth Centered Inertial (ECI) coordinate system, which does not rotate with the Earth, instead of referred to as an ECEF, as the GPS position does. Fortunately, it is possible to work in the local linearized tangent plane (LTP) without significant errors. Therefore, the LTP can be used as the global coordinate system to define both position and orientation, with *X_LTP_* pointing north, *Y_LTP_* pointing west and *Z_LTP_* pointing upward.

Therefore, IMU provides the rotation of *IMU* reference system with respect to the *LTP*. This rotation is given in terms of Euler angle roll (*ϕ*), pitch (*θ*) and yaw/heading (*ψ*), to perform subsequent rotations in the *X_LTP_Y_LTP_Z_LTP_* axes, which can be expressed by means of the Direction Cosine Matrix (DCM):

(1) $$\begin{array}{lll} R_{\text{IMU}}^{\text{LTP}} & = & R_{\psi }^{Z_{\text{LTP}}}R_{\theta }^{Y_{\text{LTP}}}R_{\phi }^{X_{\text{LTP}}}\\ & = & \left(\begin{array}{ccc} \cos\theta \cos\psi & \sin\phi \sin\theta \cos\psi -\cos\phi \sin\psi & \cos\phi \sin\theta \cos\psi +\sin\phi \sin\psi \\ \cos\theta \sin\psi & \sin\phi \sin\theta \sin\psi +\cos\phi \cos\psi & \cos\phi \sin\theta \sin\psi -\sin\phi \cos\psi \\ -\sin\theta & \sin\phi \cos\theta & \cos\phi \cos\theta \end{array}\right) \end{array}$$

Notice that the DCM of Equation (1) will also provide the global rotation of the *TOP, PRI* and *CAM* reference systems, because they were defined to have the same orientation as *IMU*.

It is well known that Euler angles present a singularity when the pitch angle (*θ*) is close to ±90° [20]. In our system, this would imply that *X_IMU_* is pointing upward or downward, *i.e.*, the box would be in the horizontal position (see Figure 4), which is not possible. Therefore, there is no need to use an alternative representation, such as quaternion.

In this way, getting the global position of *TOP* from the GNSS and the Euler angles from the IMU, it is possible to compute, for instance, the global position of the camera, *O_CAM_*, by means of the next transformation (camera orientation is given by the Euler angles):

(2) $$O_{\text{CMA}}^{\text{LTP}}=O_{\text{TOP}}^{\text{LTP}}+R_{\text{TOP}}^{\text{LTP}}\cdot d_{\text{CAM}}^{\text{TOP}}$$

Once the sensors measurements have been adapted and transformed into a common global reference system, the next step is to perform sensor fusion to improve accuracy and reliability. To that end, a Kalman filter approach can be used [12,18-20,22]. Basically, a Kalman filter corrects measurements by comparing them with their predicted values, but at the same time, predictions are corrected based on the actual measurements [20]. The idea behind this is that both estimations and observations have noise or errors. Then, a prediction model, based on the system equations, as well as an observation model have to be defined. Firstly, in the prediction step, state system variables are integrated based on the prediction model to obtain the state estimates. Secondly, in the correction step, state estimation is updated, taking into account the actual observations. These steps are repeated in a cycle.

**Figure 6.**
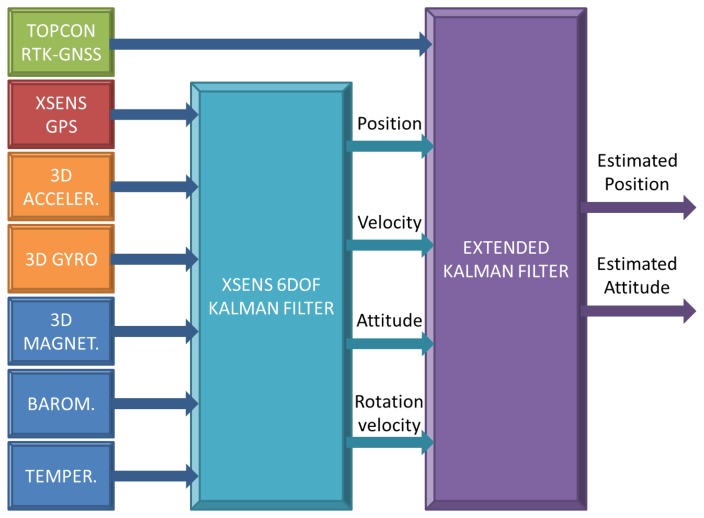
The Kalman filter structure to obtain system pose estimation (position and orientation).

Given the nonlinear nature of the proposed system, an extended Kalman filter (EKF) will be preferred to a linear Kalman filter. Furthermore, a cascaded EKF has been implemented (see Figure 6) [20]. The reasons for this configuration are threefold: first, to take advantage of the built-in Xsens Kalman filter to process inertial measurements; second, the design is more simple, and it is easier to tune; and third, even though the cascade filter is suboptimal, its performance is comparable to a full EKF [19].

The first Kalman filter is mainly devoted to integrating inertial sensors (orange blocks in Figure 6) to obtain the position and orientation estimates (prediction step). Without corrections, small errors and inaccuracies will cause the estimation errors to grow with time. Thus, in the correction step, the filter uses the stable position and velocity from the GPS (red block) to correct the estimation drift. Moreover, the orientation can also be improved from the GPS, since it is used to derive the position (see Equation (2)). Therefore, it is a loosely-coupled Kalman filter, since GPS data is used to correct IMU estimation. Finally, blue blocks are used as additional aiding sensors in the correction step. For instance, 3D magnetometers can be used to improve true north estimation based on Earth's magnetic field. The barometer is also used as an aiding sensor to measure altitude changes, increasing the vertical accuracy.

It should be noted that, as stated above, the built-in GPS has poor accuracy compared with the Topcon GR-3 RTK-GNSS, so it could seem convenient to provide corrections to the Xsens GPS to be propagated into the filter. However, it is not allowed by the device. Therefore, the second filter is mainly designed to take advantage of the high accuracy of the Topcon GR3 RTK-GNSS to estimate the system pose (position and orientation).

The EKF is based on the one proposed by Gavrilets (see [23] for details). EKF requires one to compute the derivative of the rotation matrix, which cannot be solved in closed form when using Euler angles [20]. Therefore, using the quaternion method within the EKF will be more effective. The quaternion is free of singularities, but is subject to a unit norm constraint [23]. Then, the state vector is defined as:

(3) $$x=\left[\begin{array}{ccccc} p & \upsilon & q & a_{b} & \omega_{b} \end{array}\right]$$

where *p* = [*x y z*]*^T^* is the position, *υ* = [*υ_x_ υ_y_ υ_z_*]*^T^* the velocity, *q* = [*q*_0_ *q*_1_ *q*_2_ *q*_3_]*^T^* is a unit norm quaternion representation of orientation, *a_b_* = [*a_x_b__ a_y_b__ a_z_b__*]*^T^* is the vector of acceleration bias and *ω_b_*= [*ϕ̇_b_ θ̇_b_ ψ̇_b_*]*^T^* is the vector of gyro rate bias.

It should be noted that quaternion and Euler angles are related by means of the next equations:

(4) $$\left[\begin{array}{c} q_{0}\\ q_{1}\\ q_{2}\\ q_{3} \end{array}\right]=\left[\begin{array}{ccc} \cos(\phi /2)\cos(\theta /2)\cos(\psi /2) & + & \sin(\phi /2)\sin(\theta /2)\sin(\psi /2)\\ \sin(\phi /2)\cos(\theta /2)\cos(\psi /2) & - & \cos(\phi /2)\sin(\theta /2)\sin(\psi /2)\\ \cos(\phi /2)\sin(\theta /2)\cos(\psi /2) & + & \sin(\phi /2)\cos(\theta /2)\sin(\psi /2)\\ \cos(\phi /2)\cos(\theta /2)\sin(\psi /2) & - & \sin(\phi /2)\sin(\theta /2)\cos(\psi /2) \end{array}\right]$$

(5) $$\left[\begin{array}{c} \phi \\ \theta \\ \psi \end{array}\right]=\left[\begin{array}{c} \tan^{-1}\left(\frac{2q_{2}q_{3}+2q_{0}q_{1}}{2q_{0}^{2}+2q_{3}^{2}-1}\right)\\ \sin^{-1}\left(2q_{0}q_{2}-2q_{1}q_{3}\right)\\ \tan^{-1}\left(\frac{2q_{1}q_{2}+2q_{0}q_{3}}{2q_{0}^{2}+2q_{1}^{2}-1}\right) \end{array}\right]$$

The discrete-time update equations are:

(6) $$\begin{array}{lll} p_{t+1} & = & p_{t}+{\dot{p}}_{t}\cdot dt\\ \upsilon_{t+1} & = & \upsilon_{t}+{\dot{\upsilon }}_{t}\cdot dt\\ q_{t+1} & = & \exp\left(-\frac{1}{2}\Omega \cdot dt\right)q_{t}\\ a_{b_{t}+1} & = & a_{b_{t}}+\eta_{a_{b}t}\\ \omega_{b_{t}+1} & = & \omega_{b_{t}}+n_{\omega_{b}t} \end{array}$$

where *η_a_b__* and *η_ω_b__* are noise terms and Ω is a skew-symmetric matrix composed of the gyro rate measurements and bias:

(7) $$\Omega =\left[\begin{array}{cccc} 0 & \left(\dot{\phi }-{\dot{\phi }}_{b}\right) & \left(\dot{\theta }-{\dot{\theta }}_{b}\right) & \left(\dot{\psi }-{\dot{\psi }}_{b}\right)\\ -(\dot{\phi }-{\dot{\phi }}_{b}) & 0 & -(\dot{\psi }-{\dot{\psi }}_{b}) & \left(\dot{\theta }-{\dot{\theta }}_{b}\right)\\ -(\dot{\theta }-{\dot{\theta }}_{b}) & \left(\dot{\psi }-{\dot{\psi }}_{b}\right) & 0 & -(\dot{\phi }-{\dot{\phi }}_{b})\\ -(\dot{\psi }-{\dot{\psi }}_{b}) & -(\dot{\theta }-{\dot{\theta }}_{b}) & \left(\dot{\phi }-{\dot{\phi }}_{b}\right) & 0 \end{array}\right]$$

Corrections for the Topcon GR3 RTK-GNSS, for instance, can be done based on the next lever arm equations for position and velocity [22]:

(8) $$\begin{array}{l} O_{\text{TOP}}^{\text{LTP}}=p+R\cdot d_{\text{IMU}}^{\text{TOP}}+\eta_{p}\\ V_{\text{TOP}}^{\text{LTP}}=\upsilon +R\cdot \omega \times d_{\text{IMU}}^{\text{TOP}}+\eta_{\upsilon } \end{array}$$

where *η_p_* and *η_υ_* are noise terms, *ω* is the gyro rate vector, [*ϕ̇*, *θ̇*, *ψ̇*], and *R* is the DCM of the orientation angles that in terms of the quaternion is given by:

(9) $$R=\left[\begin{array}{ccc} 2q_{0}^{2}+2q_{1}^{2}-1 & 2q_{1}q_{2}-2q_{0}q_{3} & 2q_{1}q_{3}+2q_{0}q_{2}\\ 2q_{1}q_{2}+2q_{0}q_{3} & 2q_{0}^{2}+2q_{2}^{2}-1 & 2q_{2}q_{3}-2q_{0}q_{1}\\ 2q_{1}q_{3}-2q_{0}q_{2} & 2q_{2}q_{3}+2q_{0}q_{1} & 2q_{0}^{2}+2q_{3}^{2}-1 \end{array}\right]$$

**Figure 7.**
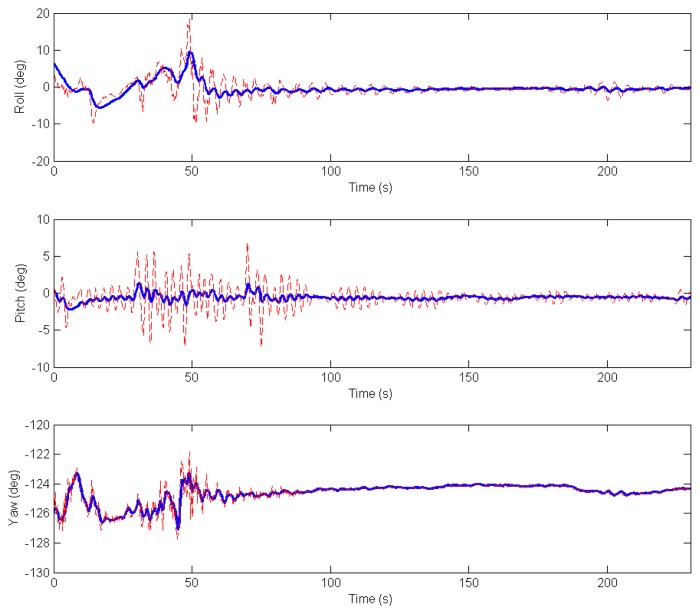
Roll, pitch and yaw filtered (blue) by the extended Kalman filter (EKF) during four minutes after deployment.

The accuracy of the estimation will be validated in the next section using a robotic total station in field experiments. Figure 7 shows the output of the filter for the orientation angles during four minutes after deployment of the system.

## 3. Experimental Results

**Figure 8.**
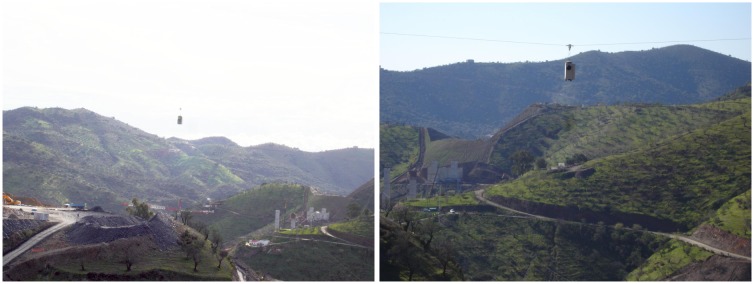
Field experiments site.

Several experiments have been conducted to test the system. These experiments were performed in a highway construction site (see Figure 8) and included, among others, the validation of the estimated position and orientation using a robotic total station, the computation of a digital surface model (DSM) and georeferencing based on camera position estimation or ground control points.

**Figure 9.**
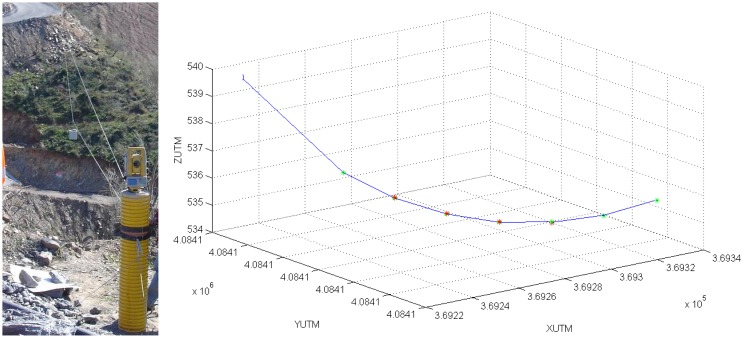
Robotic total station and measured points (red) *vs.* estimated (green) along the trajectory.

Thus, in the first experiment, seven points, located along the system trajectory, were measured with a robotic total station pointing to the on-board prism, as shown in Figure 9. The coordinates from the total station, defined in a local reference frame, were translated into UTM and compared with the prism coordinates estimated by the cascaded EKF, as shown in Table 1. The Root Mean Squared Error (RMSE) was 0.04 m and the Mean Absolute Error (MEA), 0.03 m. Notice that orientation estimations are also used to compute the prism coordinates, due to the lever arm effect.

A second experiment was devoted to collecting images in order to construct a digital surface model (DSM), *i.e.*, a model of the Earth's surface, including all objects on it, of a rough, bumpy and sloping terrain. This type of model is, sometimes, also known as a digital elevation model (DEM), while digital terrain models (DTMs) focus on the bare ground surface without any objects. There are some, very accurate, commercial applications, like the Leica Photogrammetry Suite (LPS) [24], to compute such models. However, in this paper, instead of commercial software, we made use of open applications to compute the DSM based on the emergent structure from motion (SfM) techniques [25-33].

**Figure 10.**
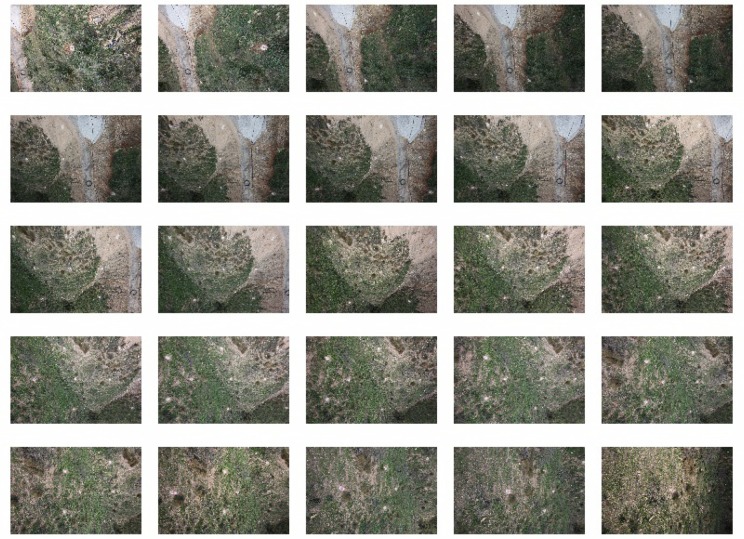
Image set used in the structure from motion approach.

Structure from motion intends to estimate a 3D structure based on a sequence of 2D images (see Figure 10). Moreover, with these techniques, it is possible to estimate both the inner and exterior orientation of the camera. The inner orientation corresponds to the camera geometry (it is defined by parameters, such as the focal length, principle point, lens distortions, *etc.*), while the exterior orientation is its position and orientation when the image was captured. Another interesting feature of these techniques is that they can be easily applied by means of open software, as will be shown below.

Some of these open software applications are mainly devoted to the determination of the camera parameters and a sparse point cloud reconstruction (VisualSFM [25], Bundler [28], APERO [31], *etc.)* while others provide dense point clouds or polygonal models (Patch-based Multi-view Stereo Software (PMVS2) [27], MicMac [32], SURE [33]…). In [34], Remondino *et al.* present a very interesting, in depth comparison of these tools based on different large and complex datasets. In this paper, we have used VisualSFM and PMVS2. VisualSFM offers a GUIfor image feature extraction, automatic matching, ground control points management and interfacing with PMVS2.

**Figure 11.**
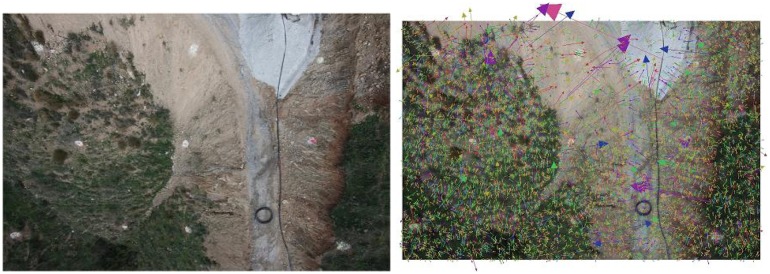
Features of interest detected in an image.

Figure 10 depicts the 25 image sequence that will be used to compute the DSM. In the first step, the software automatically extracts features that can be of interest in each image, as shown in Figure 11. Feature detection is based on the Scale Invariant Feature Transform (SIFT) [30], which is a region detector, robust with respect to rotations, translations or scales in images [35].

**Figure 12.**
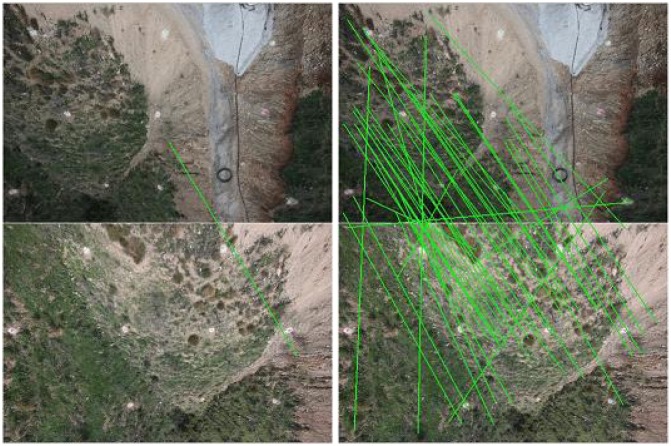
Single feature matching **(left)** and the complete set of matchings **(right)** in a given pair of images.

Secondly, an automatic matching of features in every pair of images is performed. Figure 12 shows the matching of a single feature (left) and all the features matched in a given pair of images (right). From the matched points in different images, a Bundle Block Adjustment [26,36] is performed in order to compute the inner and external orientations of the camera, which minimize the re-projection error. Generally, it is formulated as a nonlinear least-squares problem, where the error is the squared norm of the difference between the observed feature location and the projection of the corresponding 3D point on the image plane of the camera and numerically solved by using the Levenberg-Marquardt (LM) algorithm [37]. Moreover, VisualSFM implements a Multicore Bundle Adjustment [36] that exploits hardware parallelism for efficiently solving large-scale 3D scene reconstruction problems.

**Figure 13.**
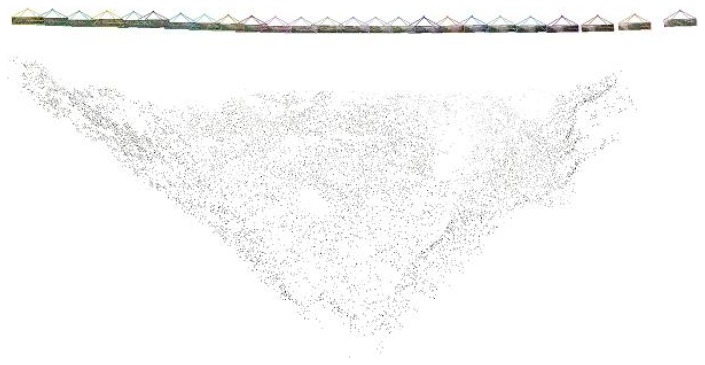
Sparse point cloud and estimated camera locations.

In this way, it is possible to obtain a 3D representation of the matched points, which is a sparse point cloud, as shown in Figure 13. This figure also depicts the 25 camera pose estimates. The sparse point cloud has 26.659 points (13.556 of them seen by more than three cameras) and 90.609 projections (65.501 seen by more than three cameras). The adjustment estimated three translations, three rotations, the focal length and one radial distortion parameter for each image, with a residual error of 1.41.

**Figure 14.**
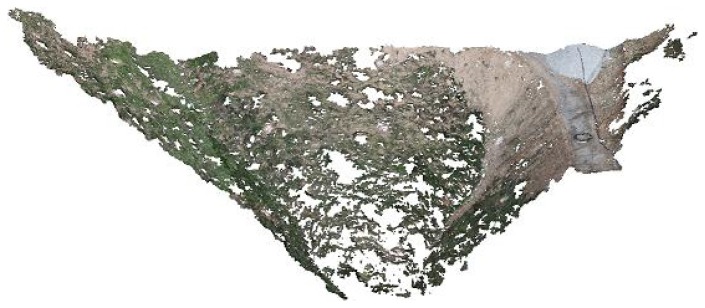
Dense point cloud representation of the experiment site.

**Figure 15.**
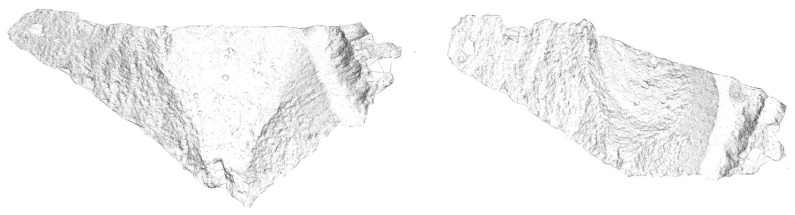
Different views of the computed digital surface model.

**Figure 16.**
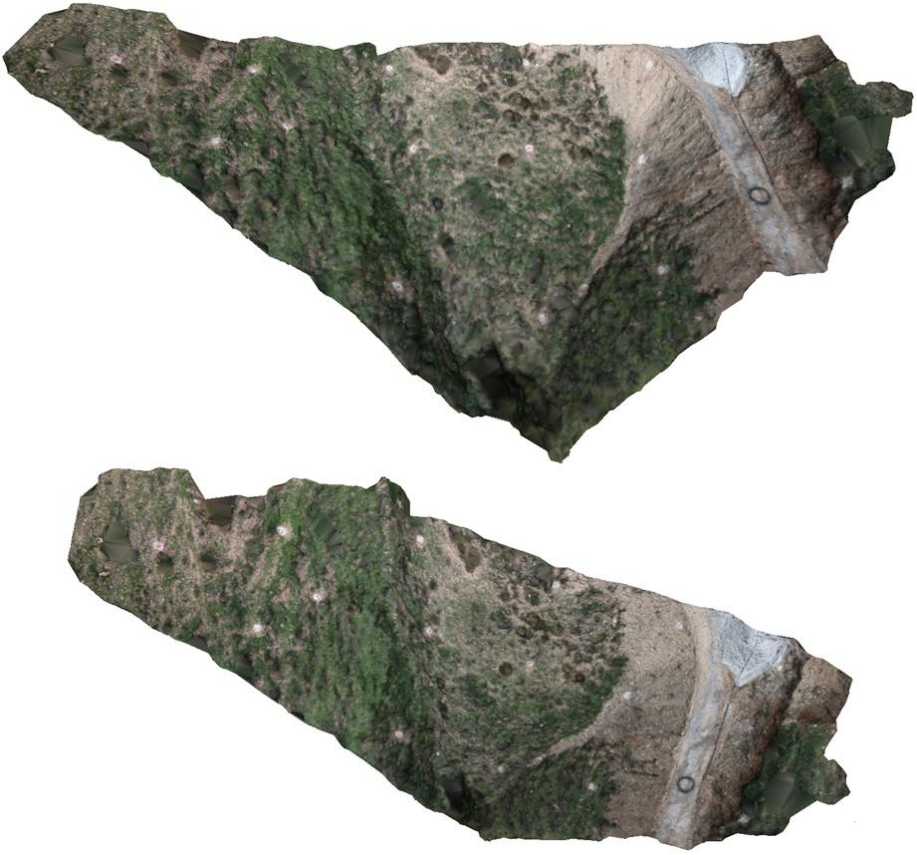
Different views of the textured digital surface model.

Moreover, it is also possible to compute a dense point cloud from the sparse one. Such a computation is performed by the Patch-based Multi-view Stereo Software (PMVS2) based on a multi-view stereo algorithm [27]. Figure 14 shows the obtained dense point cloud with a 1.217.381 vertex (the sparse point cloud had 26.659 points). The new dense point cloud can be converted into a surface by means of, for instance, a Poisson reconstruction algorithm [38], yielding a digital surface model (DSM) of the rough, bumpy and sloping terrain, as the one shown in Figures 15 and 16.

Nevertheless, the computed DSM is defined within its local coordinate system. Therefore, the final step consists of georeferencing the model, *i.e.,* the model coordinates have to be transformed into world coordinates. This can be performed, for instance, by applying Horn's method [39] to compute the optimal affine transformation (rotation *R*, translation *T* and scaling *S)* that best maps a set of points of the model, *P^M^*, into the real world, *P^W^*:

(10) $$P^{W}=S\cdot R\cdot P^{M}+T$$

Obviously, both model and world coordinates need to be known for each point in the set. To that end, it is possible to use camera coordinates or ground control points. Georeferencing by using camera coordinates measured by the system is called direct georeferencing. In this case, coordinates (in the local model and the real world) of 25 cameras were available to compute the transformation of Equation (10). Table 2 shows the camera coordinates in the local model, the ones derived from the EKF (in UTM) and the world coordinates resulting from applying the affine transformation, as well as the norm of the difference between both positions. The Root Mean Squared Error (RMSE) considering the 25 cameras is 0.23 m and the Mean Absolute Error (MAE), 0.20 m.

**Figure 17.**
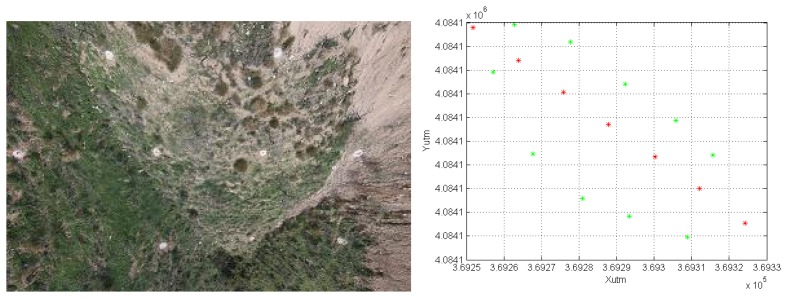
Ground control points in the experiment area.

**Figure 18.**
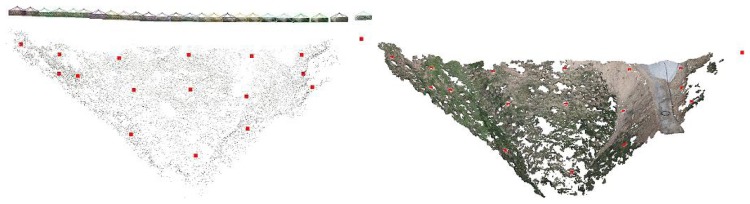
Ground control points on the sparse and dense point clouds.

Finally, another option is georeferencing based on ground control points, *i.e.*, by transforming the GCP coordinates in the local model into the GCPs in the real world. To improve performance, GCPs have to be distributed in such a way that several of them appear in consecutive overlapping images. Seventeen GCPs were signalized in the experiment area (see Figure 17), by red (7) or yellow (10) circular targets on a white background, and their coordinates recorded with a total station. VisualSFM facilitates GCP management. Thus, GCP can be manually matched directly onto the 2D images and, then, automatically converted to the 3D model, as shown in Figure 18. Then, the affine transformation that converts the seventeen GCPs from model coordinates into world coordinates is calculated, yielding approximation errors of 0.17 m RMSE and 0.13 m MAE.

## 4. Conclusions

Advances in sensor technologies have provided surveyors and construction engineers with a complete set of high accuracy ground-level tools (robotic total stations, TLS, RTK-GNSS, *etc.*) for surveying, layout and grade management of construction projects. On the other hand, improvements in digital image, CMOS camera sensors, MEMS inertial measurement units (IMU), integrated IMU-GPS devices, laser sensors, *etc.*, have produced lightweight, low-cost MEMS-based sensors that have boosted the spread of unmanned aircraft systems applied to aerial photogrammetry, surveying and engineering applications.

The counter-part of these low-cost devices is a reduction of accuracy and performance, which can be mitigated by using sensor fusion techniques for pose estimation. Moreover, these vehicles usually have a reduced payload (lower than 3 kg), limited autonomy and even require skilled operators, either to remote control or as safety pilots in the case of UAVs with autopilot systems (in some cases, the autopilot can only be activated when the vehicle is in the air, but take-off and landing have to be operated manually). UAVs without these limitations have a significantly higher cost.

This paper has presented a new system designed in a research project, funded by the Spanish construction company, Sacyr SAU, to take advantage of ground device accuracy (facilitating integration with surveying total stations, RTK-GNSS and the construction site infrastructure) and, at the same time, overcoming the limitations of aerial vehicles with respect to weight, autonomy and the need for skilled operators. Thus, the system integrates an accurate RTK-GNSS, commonly used at construction sites, a prism for interfacing with a robotic total station, a high resolution camera and a medium-cost IMU-GPS, into a device, weighing around 12 kg in total. It moves between two mounting points, in a blondin ropeway configuration, at the construction site, taking pictures and recording position and orientation data along the cable path. Using two mounting points can be a disadvantage with respect to the coverage area, but this could be solved by using a Skycam configuration.

A cascaded extended Kalman filter is used to integrate measurements from the on-board inertial measurement unit (IMU), a GPS and a RTK-GNSS, which has been validated with a robotic total station in field experiments in a construction site, with an error of 0.03 m (MAE).

Finally, instead of commercial software, in this paper, we have made use of open software to compute a digital surface model (DSM) of a rough, bumpy and sloping terrain. Namely, structure from motion (SfM) techniques have been applied to obtain the 3D model from a sequence of 25 images. Among the available software, VisualSFM was selected, because its GUI facilitates image feature extraction, automatic matching, ground control points management and interfacing with PMVS2 (for dense point cloud calculation). The Bundle Block Adjustment resulted in a sparse point cloud with 26.659 points (13.556 of them seen by more than three cameras). On the other hand, the obtained dense point cloud had a 1.217.381 vertex. The georeferencing of the DSM was performed based on the estimated camera positions, with an approximation error of 0.20 m (MAE), and also using 17 ground control points (GCP), with an error of 0.13 m (MAE). Even if these results are not accurate enough for certain applications, they can be considered good, because the slopes of the terrain and the fact that the software was used as is, without particular improvements. However, this should be tested with larger sequences of images and different terrains.

## Figures and Tables

**Table 1.** Position of the on-board prism measured with the total station (TS) in Universal Transverse Mercator (UTM) coordinates, estimated by the Kalman filter (EKF) and error.

**Point**	***X****_TS_*	***Y****_TS_*	***Z****_TS_*	***X*_*EKF*_**	***Y*_*EKF*_**	***Z*_*EKF*_**	**Error (m)**
1	369,251.77	4,084,123.89	536.37	369,251.77	4,084,123.89	536.37	0.01
2	369,263.71	4,084,117.20	535.56	369,263.72	4,084,117.20	535.54	0.03
3	369,275.59	4,084,110.31	535.08	369,275.60	4,084,110.32	535.05	0.03
4	369,287.86	4,084,103.47	534.88	369,287.84	4,084,103.43	534.86	0.05
5	369,299.87	4,084,096.54	534.93	369,299.88	4,084,096.57	534.97	0.06
6	369,311.94	4,084,089.74	535.29	369,311.94	4,084,089.73	535.30	0.02
7	369,324.05	4,084,082.69	535.97	369,324.06	4,084,082.71	535.98	0.03

**Table 2.** Camera coordinates in the local model (L), UTM estimated by the Kalman filter (EKF), results from the transformation (T) and error.

***X****_L_*	***Y****_L_*	***Z****_L_*	***X****_EKF_*	***Y****_EKF_*	***Z****_EKF_*	***X_t_***	***Y****_T_*	***Z_t_***	**Error (m)**
7.954	0.007	−0.162	369,319.94	4,084,085.17	535.76	369,319.89	4,084,085.23	535.52	0.25
6.381	0.029	−0.122	369,315.74	4,084,087.54	535.52	369,315.68	4,084,087.55	535.33	0.20
4.884	0.024	−0.109	369,311.74	4,084,089.84	535.34	369,311.71	4,084,089.83	535.23	0.11
3.537	0.007	−0.112	369,308.14	4,084,091.87	535.21	369,308.16	4,084,091.91	535.19	0.05
2.255	−0.043	−0.115	369,304.68	4,084,093.89	535.13	369,304.83	4,084,093.98	535.14	0.18
1.045	−0.047	−0.132	369,301.51	4,084,095.93	535.04	369,301.62	4,084,095.82	535.14	0.19
0.019	−0.027	−0.129	369,298.99	4,084,097.41	535.00	369,298.86	4,084,097.32	535.09	0.18
…	…	…	…	…	…	…	…	…	…
